# Dramatic Response to First Line Single Agent Pembrolizumab in Anaplastic Thyroid Carcinoma

**DOI:** 10.1155/2019/9095753

**Published:** 2019-11-26

**Authors:** Valérie Spalart, Barbara Legius, Kurt Segers, Johan Coolen, Brigitte Maes, Lynn Decoster

**Affiliations:** ^1^AZ Turnhout, Department of Internal Medicine, Turnhout, Belgium; ^2^AZ Turnhout, Department of Pulmonology/Respiratory Oncology, Turnhout, Belgium; ^3^AZ Turnhout, Department of Pathology, Turnhout, Belgium; ^4^University Hospitals Leuven, Department of Radiology, Leuven, Belgium; ^5^Jessa Hospital Hasselt, Laboratory for Molecular Diagnostics, Department of Clinical Biology, Hasselt, Belgium

## Abstract

Anaplastic thyroid carcinoma (ATC) is a deadly disease with very limited therapeutic options. There is an urgent need for new and efficacious drugs. Unfortunately accrual in clinical trials is problematic because of the rarity of the disease and often poor performance status at diagnosis. Recently some data have emerged suggesting a role for immunotherapy in the treatment of ATC. We describe the case of a 75-year-old patient with poor performance status and compromised airway and oesophagus at diagnosis, showing a rapid and dramatic response to first line single agent pembrolizumab. Disease progression in the brain occurred 16 months after initial diagnosis. At that time there was ongoing extracranial disease control.

## 1. Introduction

Anaplastic thyroid carcinoma (ATC) is a rare and aggressive disease representing 1-2% of all thyroid cancers but accounting for 50% of annual thyroid cancer related mortality. Approximately 50% of patients present with metastatic disease and treatment options are limited except for a subset of patients with BRAF-mutated tumors. Recent data suggest a potential role for immunotherapy in this disease desperate for new therapies. We describe a patient with poor performance status at presentation showing a rapid and dramatic response to first line single agent pembrolizumab.

## 2. Case Presentation

A 75-year-old female never smoker presented to the ear, nose and throat specialist with hoarseness, dry cough, difficulty swallowing and a 3 kg weight loss since 1 month.

5 years earlier she was treated with radioactive iodine for a toxic multinodular goiter. She also had a past medical history of a stage *Ic* well-differentiated serous papillary ovarian carcinoma treated with surgery and adjuvant chemotherapy.

**Figure 1:**
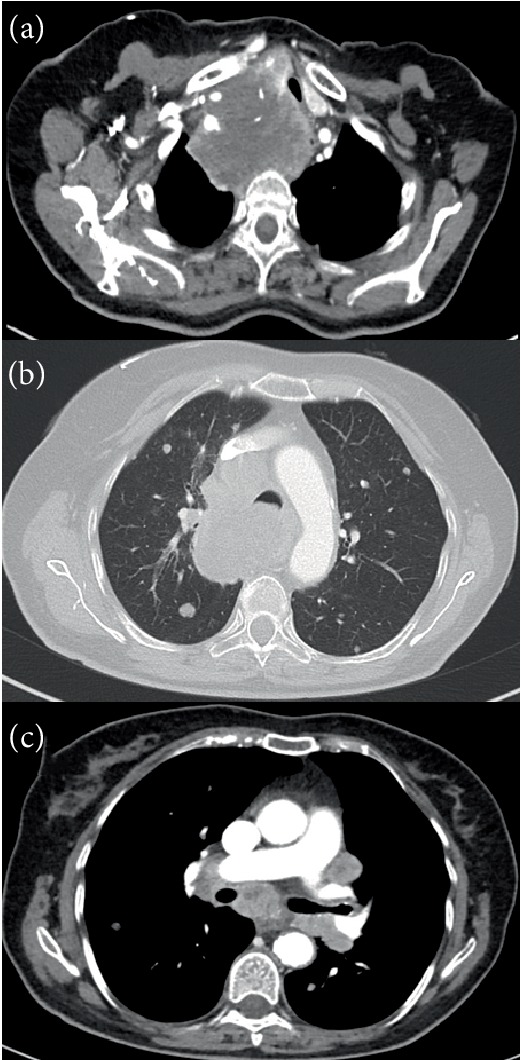
Baseline chest CT showing a large mass originating from the right thyroid lobe (a), with lung metastases (b), and hilar and mediastinal lymph nodes (c). (a), (c) images in mediastinal window. (b) image in lung window.

On clinical examination, a multinodular and enlarged thyroid gland was palpated. A computed tomography (CT) scan of the neck and chest revealed a large mass (8 × 9 cm) in the anterior mediastinum involving the right thyroid lobe and causing deviation of both trachea and oesophagus. There were multiple pathologically enlarged cervical, supraclavicular and mediastinal lymph nodes as well as multiple bilateral lung metastases (Figure 1). Further staging revealed a bone metastasis in the right iliac crest. During bronchoscopy paralysis of the right vocal cord was noticed as well as invasion of the trachea with reduction of tracheal diameter estimated at 25%. No intracranial metastases were present on baseline brain CT imaging. A biopsy of a cervical lymph node was performed showing diffuse infiltration by an abnormal epithelial cell proliferation. The abnormal cells were large with large and hyperchromatic nuclei. Multiple mitotic figures were present as well as necrosis and several infiltrating lymphocytes. Immunohistochemical studies on the neoplastic cells were negative for thyroid transcription factor-1 (TTF-1), PAX-8, thyroglobulin, cytokeratin AE1/AE3, cytokeratin 7, CD 45, CD 34 and S-100. The pathological findings were compatible with an anaplastic large cell malignancy.

**Figure 2:**
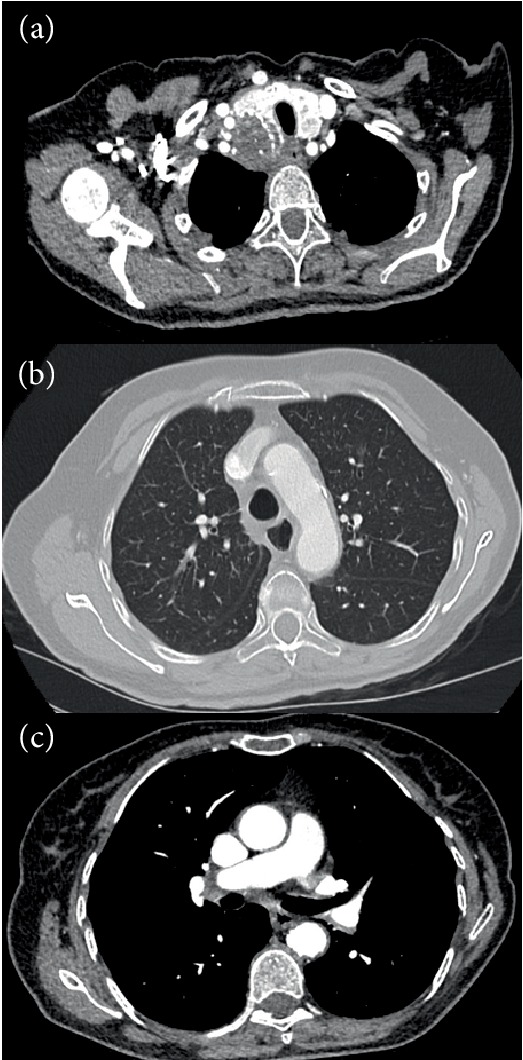
Chest CT after 3 cycles of pembrolizumab showing marked regression of primary tumor (a), lung metastases and (b), lymph nodes (c).

The case was discussed at the multidisciplinary tumor board and based on clinical presentation, imaging and pathology a diagnosis of primary anaplastic thyroid cancer was made. Next generation sequencing (NGS, TRUSEQ, MISEQ, ILLUMINA) was negative for molecular drivers. PDL1-staining was positive with a high expression (60% of tumor cells). At that point the clinical condition of the patient was rapidly deteriorating due to dysphagia with impaired food intake. The possibility of palliative stenting of the oesophagus was discussed with the gastroenterologist but was considered technically impossible. The patient was unfit for a clinical trial. Based on occasional case reports describing exceptional response to immunotherapy in patients with anaplastic thyroid cancer, the patient was offered the possibility of treatment with pembrolizumab. After only 2 cycles, the patient noticed a marked improvement of dysphagia and after 3 cycles, a near complete response was seen on CT (Figure 2). Thyroid function was thoroughly monitored during treatment and remained normal at all times.

After 8 cycles however, the patient developed a severe grade 4 colitis (bloody stools, severe dehydration with hypotension) requiring aggressive fluid resuscitation and treatment with systemic steroids. Due to further clinical deterioration infliximab was administered, after which gradual improvement occurred. 3 months after the last administration of pembrolizumab, there were no signs of tumor progression. As most guidelines recommend permanent discontinuation of immunotherapy after grade 4 gastro-intestinal toxicity, a watchful waiting approach was proposed with follow-up CT of the chest and neck every 3 months.

**Figure 3:**
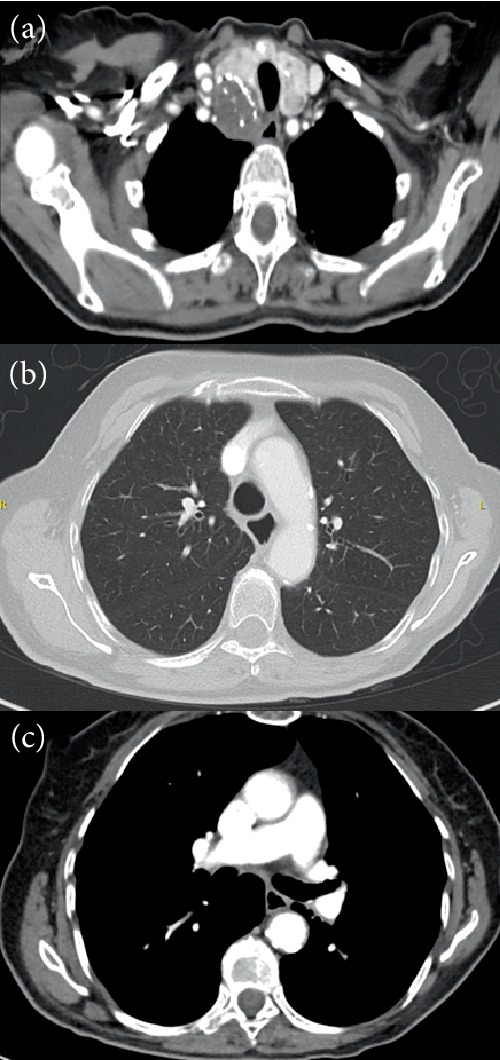
Chest CT 6 months after start of pembrolizumab showing ongoing response.

6 months later (16 months after diagnosis), the patient presented to the emergency department with gait difficulties, falling tendency to the left, left-sided facial palsy and hypoesthesia of the left upper arm. Magnetic resonance imaging (MRI) of the brain revealed a 19 × 18 × 19 mm lesion in the right thalamus. Stereotactic biopsy of this lesion was compatible with metastasis of known anaplastic large cell carcinoma. There were no signs of extra-cranial tumor progression (Figure 3). Stereotactic radiotherapy to the brain lesion was performed. However her neurological condition did not improve. Because of poor performance status, previous grade 4 gastro-intestinal toxicity and mainly intracranial treatment failure, a re-challenge with pembrolizumab was not considered nor was second line chemotherapy. The patient was referred for hospice care. She died 18 months after initial diagnosis.

## 3. Discussion

Anaplastic thyroid carcinoma (ATC) is a rare and aggressive disease with a median overall survival of less than 6 months and a 1-year survival of only 20%. In metastatic disease particularly, treatment options are limited [1]. The evidence for conventional chemotherapy is scarce as large randomized trials are lacking. Taxanes, doxorubicin and platin have shown some activity either as single agent or in combination, with response rates between 15% and 25% [2].

Lately promising results were reported with targeted therapies. In a single-arm, open-label study 17 ATC-patients were treated with the multikinase-inhibitor lenvatinib. The objective response rate was 24%, the median progression-free survival (PFS) was 7.4 months and the median overall survival (OS) 10.6 months [3]. More importantly, in BRAF-mutated tumors, the combination of the selective kinase inhibitors (dabrafenib and trametinib) achieved a response rate of 69% and a 1-year survival of 80% [4]. Unfortunately resistance to these targeted therapies inevitably occurs.

The role of immunotherapy in ATC remains to be elucidated. Kolllipara et al. described an exceptional response with immunotherapy (nivolumab) in a patient with BRAF positive ATC who showed a mixed response to the BRAF-inhibitor vemurafenib [5]. In a small retrospective series of salvage pembrolizumab added to a kinase inhibitor at time of progression, partial response rate was 42% with a median OS from the start of pembrolizumab of 6.9 months [6]. Little is known about the role of single agent immunotherapy. In 30 ATC patients treated with the PD1-inhibitor spartalizumab, partial response was observed in less than 20% [7]. On the contrary, Khan et al. reported 2 out of 4 partial responses in ATC patients treated with pembrolizumab [8]. Currently 2 clinical trials are examining the role of immunotherapy-only in ATC patients (pembrolizumab in NCT02688608; nivolumab plus ipilimumab in NCT03246958).

In most ATC-trials however, accrual is hampered by the rarity of the disease and the often poor performance status at initial diagnosis. In a review article on diagnosis and management of ATC, Chintakuntlawar et al. state that it might therefore be reasonable to consider PD-1 inhibitors in these patients [1]. Occasional case reports might further build the evidence for the use of immunotherapy in a disease desperate for new treatment options.

We hereby describe a case of metastatic ATC treated with single agent pembrolizumab as first line therapy. Our case illustrates that a rapid and clinically meaningful response can be obtained even in a very sick patient with compromised airway and oesophagus. Clinical benefit occurred after only 2 cycles of pembrolizumab and near complete response was obtained after 3 cycles. Unfortunately, Pembrolizumab had to be discontinued because of grade 4 colitis and relapse occurred in the brain 14 months after initial diagnosis. At that moment there was ongoing extra-cranial disease control. As the initial impressive response to immunotherapy in our patient is intriguing we retrospectively assessed tumor infiltrating lymphocytes (TILs) on H&E slides from a metastatic lymph node. Stromal TILs (sTILs) could not be scored as no stroma was present in the biopsies. The percentage of intratumoral infiltrating lymphocytes (iTILS) was 30%. Whether PD-L1 or H&E based assessment of TILs (and at which cut-off) are predictive biomarkers in anaplastic thyroid cancer is a question that hopefully can be answered by ongoing or future clinical trials.
